# Reduced tibial strain-shielding with extraosseous total knee arthroplasty revision system

**DOI:** 10.1016/j.medengphy.2018.09.006

**Published:** 2018-12

**Authors:** Tomas A. Correa, Bidyut Pal, Richard J. van Arkel, Felice Vanacore, Andrew A. Amis

**Affiliations:** a Biomechanics Group, Mechanical Engineering Department, Imperial College London, London SW7 2AZ, UK; b School of Engineering, University of Portsmouth, Portsmouth PO1 3DJ, UK; c Department of Orthopaedics and Traumatology, Marche Polytechnic University, Ancona, Italy; d Musculoskeletal Surgery Group, Department of Surgery and Cancer, Imperial College London School of Medicine, London W6 8RF, UK

**Keywords:** Revision total knee arthroplasty, Stress strain shielding, Prosthesis design, Cortical bone fixation plate, RTKA, Revision total knee arthroplasty, TKA, Total knee arthroplasty, CT, X-ray Computed tomography, PMMA, Poly methylmethacrylate, DIC, Digital image correlation

## Abstract

**Background:**

Revision total knee arthroplasty (RTKA) has poorer results than primary total knee arthroplasty (TKA), and the prostheses are invasive and cause strain-shielding of the bones near the knee. This paper describes an RTKA system with extracortical fixation. It was hypothesised that this would reduce strain-shielding compared with intramedullary fixation.

**Methods:**

Twelve replica tibiae were prepared for full-field optical surface strain analysis. They were either left intact, implanted with RTKA components with cemented intramedullary fixation stems, or implanted with a novel design with a tibial tray subframe supported by two extracortical fixation plates and screw fixation. They were loaded to simulate peak walking and stair climbing loads and the surface strains were measured using digital image correlation. The measurements were validated with strain gauge rosettes.

**Results:**

Compared to the intact bone model, extracortical fixation reduced surface strain-shielding by half versus intramedullary fixation. For all load cases and bone regions examined, the extracortical implant shielded 8–27% of bone strain, whereas the intramedullary component shielded 37–56%.

**Conclusions:**

The new fixation design, which offers less bone destruction than conventional RTKA, also reduced strain-shielding. Clinically, this design may allow greater rebuilding of bone loss, and should increase long-term fixation.

## Highlights

• A novel extracortical support system for revision of failed knee prostheses.
• Shown to reduce metaphyseal stress-shielding versus intramedullary stem fixation.
• Reduces bone loss and enables bone grafting of defects after implant loosening.
• Enables use of conventional prosthesis in a revision scenario.

## 1 Introduction

Revision total knee arthroplasty (RTKA) surgery is a growing socioeconomic burden [1], [2], and is performed at a rate of 5–11% of total primary TKA operations [3], [4]. The major causes of implant revision include mechanical loosening, infection, osteolysis, instability, and misalignment [5]. The primary objectives of RTKA surgery are pain relief, joint stabilisation, re-establishment of the anatomical joint line, restoration of bone stock, and ultimately a prompt return to full weight-bearing function [6], [7].

At the time of revision knee surgery, there is commonly some degree of bone loss from either or both the distal femur and proximal tibia [8], [9], [10]; consequently, revision implant components are almost always augmented with intramedullary stems to provide additional stability. In the case of severe bone loss, these intramedullary components can be used in combination with ‘augment’ blocks, wedges, sleeves, or allograft to fill the defects [5], [7], [8], [11], [12]. Stems are often provided in a set with options for length and cross-section available to the surgeon [9], with longer stems being frequently used to improve initial implant stability [13], [14]. However, long stems require removal of healthy bone from the medullary canal wall and, because they transfer load to the cortex away from the joint line, lead to strain-shielding of bone close to the joint line. Such strain-shielding may lead to long-term bone resorption, implant loosening and ultimately costly and higher-risk re-revision surgery [15], [16].

Extracortical fixation plates, similar to those used in fracture fixation, may present an alternative to the use of long intramedullary stems when increased stability is required due to bone loss. Plates and screws for fixation of articulating components have been used in a number of tumour prostheses [17], [18], [19]. For a revision knee replacement, this type of fixation when compared with an intramedullary stem could offer several benefits: reduced strain-shielding [20]; elimination of end-of-stem pain [21], [22] or fracture [23]; no need for the removal of healthy bone adjacent to the medullary canal; and easier removal if required. Further, the extracortical assembly could be designed to support a primary TKA component during revision, potentially reducing inventory and expense. Thus, the feasibility of an extracortical revision system warrants investigation, and advantages and disadvantages with respect to conventional intramedullary fixation must be quantitatively assessed. Given that a reduction in strain-shielding is the primary benefit considered, due to its impact on component survivability, this must be assessed prior to further concept development.

The aim of the present study was to quantify strain-shielding of proximal tibial bone by RTKA tibial components, with additional fixation provided by either (a) a conventional intramedullary stem, or (b) medial and lateral extracortical plates. It was hypothesised that the extracortical fixation would reduce bone strain-shielding when compared to conventional intramedullary RTKA components.

## 2 Methods

### 2.1 Implant component design

**Fig. 1:**
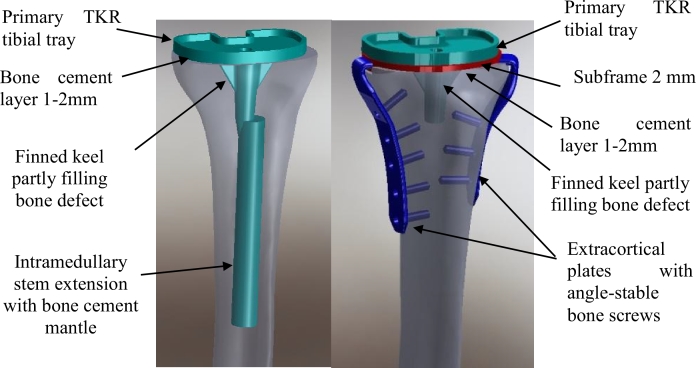
Representative CAD images of the implants used: (a) tibial component with cemented intramedullary stem fixation and (b) tibial component with medial and lateral extracortical plate fixation. Labels highlight the cementing technique.

The cemented intramedullary implant (Fig. 1(a)) comprised a PFC® Sigma® Fixed Bearing tibial tray (DePuy Orthopaedics, Inc., Warsaw, IN, USA) size 3: 47 mm anterior-posterior by 71 mm medial-lateral, with a stainless steel stem extension (14 mm diameter, 115 mm length) attached through the provided internal screw thread within the tray's short stem. Both the diameter and length were typical of medium-sized femoral and tibial stem extensions currently provided by major knee replacement manufacturers.

The extracortical implant fixation system (Fig. 1(b)) was comprised of four major components and eight screws. A titanium alloy (Ti6Al4V) sub-frame was placed below the same PFC® Sigma® Fixed Bearing tibial tray. Two Ti6Al4V extracortical plates were also attached to this sub-frame, one located medially and one antero-laterally, through mating features at the proximal ends (one protruding boss on each plate and two corresponding sockets in the sub-frame). These plates matched the longitudinal curvature of the bone, were 16 mm wide and 3 mm thick, and extended approximately 95 mm (medial) and 80 mm (lateral) distal to the joint line. Five steel screws 6 mm diameter (M6 thread) were used to attach the medial plate to the adjacent synthetic bone cortex, and three screws were used similarly on the lateral side. The screws used distally were 25 mm in length, and those used proximally (two on the medial plate and one on the lateral plate) were 20 mm in length so as not to interfere with the stem and keels of the tibial component. The screw holes in the plates were tapped with a 6 mm diameter (M6) thread, thereby mimicking angle-stable locking screw/plate technology, as commonly used in modern fracture fixation products.

### 2.2 Simulation of bone defects and implantation technique

Twelve composite synthetic tibia bone models (medium left fourth-generation [model 3401], Sawbones AG, Sweden) were prepared with a proximal tibial cut using a surgical saw. The position and orientation of the cut was defined by a custom-made cutting guide based on the geometry of the composite bone model, which was digitized from computed tomography (CT) images, to ensure a near-identical cut for all bones. Similar patient specific instruments have achieved repeatable implantation within 2° and 1 mm in synthetic knee joints [24]. The cut was oriented with a 3° posterior slope relative to a plane normal to the bone's anatomical axis, and was located 9 mm distal to the outer rim of the lateral articulating surface of the tibial plateau. Four ‘intact’ specimens were left unimplanted, to provide the baseline surface strain results from which evaluation of the strain-shielding behaviour of the two implant designs was performed.

A second stage of bone removal was performed for the remaining eight synthetic bones, to simulate the bone lost during the removal of a keeled tibial tray component (such as the aforementioned PFC® Sigma® design), including a moderate amount of bone loss adjacent to such a component. This was performed by: first, drilling a hole through to the medullary canal, simulating a previous use of the intramedullary alignment technique for primary component implantation; and second, removing all bone within 5 mm of the primary component's keel to a depth of 25 mm below the resection plane using a hand-drill and a separate custom guide block. This represented removal of the keeled primary TKA component plus the layer of bone cement around it, plus several mm of adherent cancellous bone, and/or a cavity that had become resorbed and replaced by fibrous tissue after loosening of the fixation.

Finally, four of the specimens with simulated bone loss were implanted with the conventional cemented intramedullary tibial revision implant design, and the other four were implanted with a novel tibial component with extracortical plate fixation. For the intramedullary design, poly (methyl methacrylate) (PMMA) bone cement was used between the base of the tray and the resected ‘bone’ (a 1–2 mm layer covering the entire area of the mating surfaces), to fill the areas of simulated bone defect, and between the stem and the widened medullary canal of the ‘bone’ (a 1–2 mm layer adjacent to the entire surface area of the stem) [25], [26]. For the extracortical design, PMMA was applied between the proximal cut surface of the tibia and the sub-frame, and between the sub-frame and the tibial tray (with both of these cement layers being 2 mm thick and covering the entire areas of the respective mating surfaces), and to fill the areas of simulated bone defect.

### 2.3 Loading and strain measurement

Digital image correlation (DIC), an optical technique, was used to determine bone surface strain patterns under axial compressive loading, to assess the strain-shielding behaviour of the implants [27], [28]. It combines the advantages of a conventional in-vitro strain gauge experiment (such as realistic contact mechanics between the implant components and at the bone/implant interface) with the full-field strain measurements akin to the predictions obtainable from finite element models.

High-contrast speckle patterns were applied to the most-proximal 200 mm of each synthetic bone, using matt paint. The distal ends were potted in a 60 mm diameter by 100 mm long cylinder with PMMA, and mounted with the proximal resection plane aligned horizontally, which corresponded to the mechanical axis of the tibia having the same orientation as the electromechanical testing machine's loading axis.

**Fig. 2:**
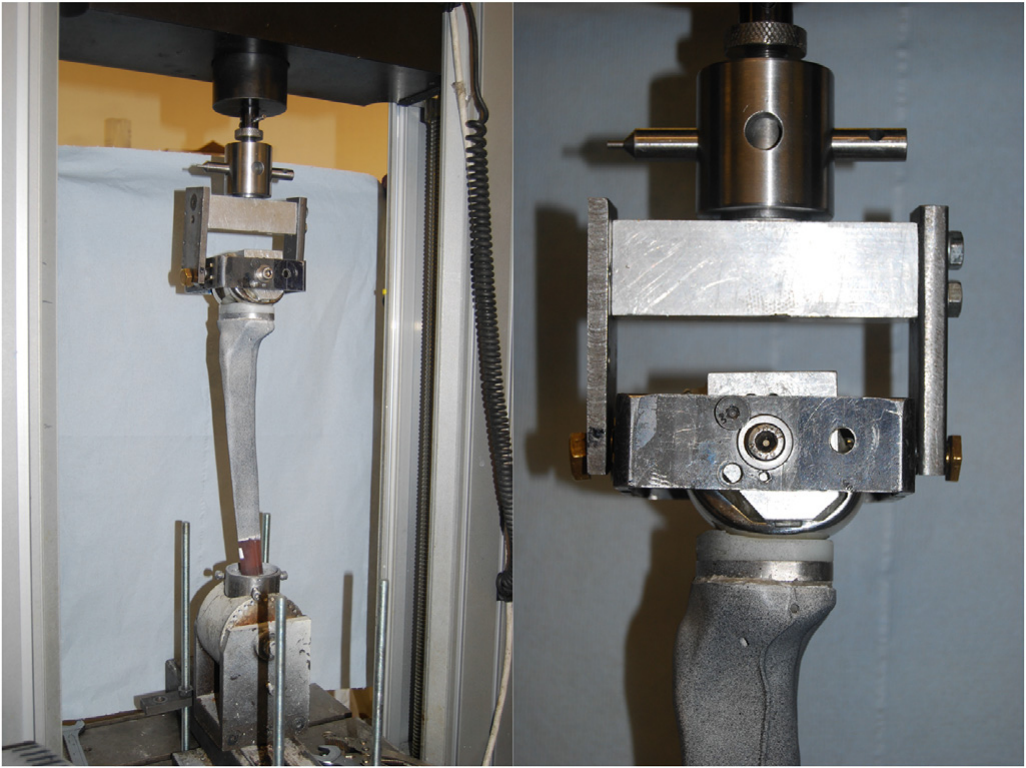
The testing set-up. The mounting frame around the femoral component is attached to the loading frame above it by bearings that allow freedom to rotate in ab-/adduction; the medial-lateral position of this pivot was located to create the desired % load on each of the femoral and tibial condyles. (Left) full setup view; (right) lateral aspect.

Synthetic tibiae were loaded through a femoral component and the tibial tray's corresponding XLK™ cross-linked polyethylene insert (DePuy Orthopaedics, Inc., Warsaw, IN, USA) with the knee in extension, using a single-axis electromechanical materials testing machine (Model 5866, Instron, High Wycombe, UK) with a 10 kN load cell. Four loading conditions were applied to each tibia: gait with a balanced mediolateral load, gait with a medially-weighted load, stair ascent with a balanced load and stair ascent with a medially-weighted load (Table 1). Peak load magnitudes for gait (3.0 BW) and stair ascent (3.6 BW) were based on published values [29], [30], and on in vivo loading data from the database Orthoload [31]. Medial load-weighting was controlled through the medial-lateral position of a pivot axis between the condyles of the custom femoral component fixture; this fixture allowed for the medial-weighting to be controlled with a maximum error of 2%. During gait and stair ascent, the peak load vector direction is between 1 and 6° anteriorly/proximally depending on the patient [31], and therefore no anterior component of load was applied during testing. Tibial bending stresses will be influenced by the knee contact point in the sagittal plane. In vivo measurements suggest that the position and trends vary between patients, implant designs and the degree of weight bearing [32]. The testing set-up simulated a weight-bearing posterior-stabilized knee (Fig. 2), for which the data suggest that there is little change in contact point with flexion, being positioned between 4 and 5 mm posteriorly from the centre of the tibial tray in the sagittal plane [32]. The tibial mounting was adjusted such that, when the femoral component was lowered into weight-bearing engagement with the PE insert, it did not cause anterior-posterior deflection of the tibia.

**Table 1:** Four loading conditions used for experimental measurement of longitudinal strain.

	Medial load	Medial	Lateral
	weighting (%)	load (kN)	load (kN)
**Gait (3.0** **×** **BW = 2.03** **kN)**	70	1.42	0.61
	50	1.02	1.02
**Stair ascent (3.6** **×** **BW = 2.47** **kN)**	70	1.73	0.74
	50	1.24	1.24

Three loading cycles with the greatest peak load (2.47 kN) were applied prior to strain measurement to allow the implant system to “bed in” to the synthetic bone. All loads were applied at a rate of 1 mm/min and the gait and stair ascent peak loads were held for 5 s before strain measurement. Two repetitions per loading condition were performed for each condition.

Throughout loading, the speckle pattern features were imaged by two charge-coupled DIC cameras with 50 mm lenses, located 1.3 m from the bone, providing a 210 mm × 175 mm field of view and a depth of focus field of 165 mm. Calibration was performed using a 175 × 140 mm panel. The medial, lateral, and posterior surfaces of the bone were imaged separately. Displacements and strains were calculated using an ARAMIS 5 M software system (GOM mbH, Braunschweig, Germany), and consistent coordinate systems were generated for each surface (medial, lateral and posterior) based on common landmarks.

### 2.4 Data analysis

Compressive strains were reported as positive values. Pilot testing showed that the minimum principal strains on the areas under greatest compression (typically postero-medial) were generally below 0.20% (2000 microstrain), and that transverse strain and shear strain were of much lower magnitude than the longitudinal strain in the direction of compressive loading. Therefore, similar to Sztefek et al. [28] and Zimmermann et al. [33], only the longitudinal component of the strains was used for strain analysis as the small values of transverse and shear strain were affected by noise when the field of view was enlarged to allow simultaneous measurement of strains adjacent to the tibial plateau and also distal to the tip of the long cemented intramedullary stem. Strain fields were analysed in three longitudinal regions: a proximal region 0–90 mm from the resection plane (corresponding to the length where the medial extracortical plate could be placed), a middle region (90–150 mm), corresponding to the length where the stem could be present but not the extracortical plate, and a distal region (150–200 mm) where neither stem nor plates would be present.

Due to the orientation, shape and loading of the synthetic bone specimens during the experiment, which simulated the effects of the knee adduction moment during gait and stair ascent, large areas of both the medial and posterior surfaces were placed under axial compression, while the lateral surfaces were largely uncompressed; therefore, results are only presented for the medial and posterior faces, where the largest differences in compressive strains were observed. The mean percentage strain-shielding caused by the implants compared to the intact case was quantified by finding the mean strain along two repeatable proximal-distal lines parallel to the anatomical axis: the first on the medial surface was located 30 mm posterior to the anterior crest, and the second on the posterior surface was located 5 mm medial to the surface centreline. These lines were chosen based on pilot testing, which confirmed that the data obtained were repeatable with low noise.

### 2.5 Strain measurement technique comparison

Stereo-DIC-measured strains can be sensitive to the speckle pattern design and the system set-up (lighting, focal lengths, camera spacing, calibration, etc.). To ensure that the experimental set-up used in this experiment produced meaningful results, one synthetic bone specimen was prepared with six pre-wired 120Ω strain gauge rosettes (3 mm 0°/45°/90° grid, KFH-3-120-D17-11L1M2S, Omega Engineering Ltd, Manchester, UK) using a cyanoacrylate adhesive in accordance with the manufacturer's instructions. These gauges were mounted in regions of low strain gradient on the anteromedial and posterior faces of the tibia replicating an established protocol [34]. The gauges were wired directly to a 40-channel data logger (FE-MM40, Fylde Electronic Laboratories Ltd., Preston, UK) which recorded strains during loading tests. The area of bone immediately surrounding these strain gauges was then prepared with the DIC speckle pattern to verify the magnitude of strains recorded by this specific DIC set-up. The longitudinal strains for the DIC method were then averaged for the area of bone surrounding the gauge and compared to the middle gauge of the rosette (orientated on the longitudinal axis).

### 2.6 Statistical analysis

Stair climbing data with 70% medially weighted load was used for the statistical analysis as this loading scenario put the posterior and medial surfaces under the greatest strain due to the increased load. Raw micro-strain data were tested for normality with a Shapiro–Wilk test and then analysed using SPSS software (version 22, SPSS Inc., Chicago, Illinois) with a two-factor analysis of variance (ANOVA): the independent variables were implant state (intact, intramedullary and extracortical) and distance from the tibial plateau resection (20–180 mm in 20 mm intervals). The dependent variables (two separate analyses) were the measured longitudinal strains on the medial and posterior surfaces. Post-hoc *t*-tests with Bonferroni correction were applied when differences across tests were found. The significance level was set at *p* < 0.05. Adjusted *p*-values, multiplied by the appropriate Bonferroni correction factor in SPSS, have been reported rather than reducing the significance level.

For each measurement taken in the proximal and middle regions, the strain-shielding caused by an implant for an average specimen was also calculated as the difference between the mean intact and mean implanted surface strains as a percentage of the mean intact strains. Two unpaired equal variance *t*-tests were then applied (i.e., for the posterior and medial surfaces) to detect differences in strain-shielding for an average implant.

## 3 Results

**Fig. 3:**
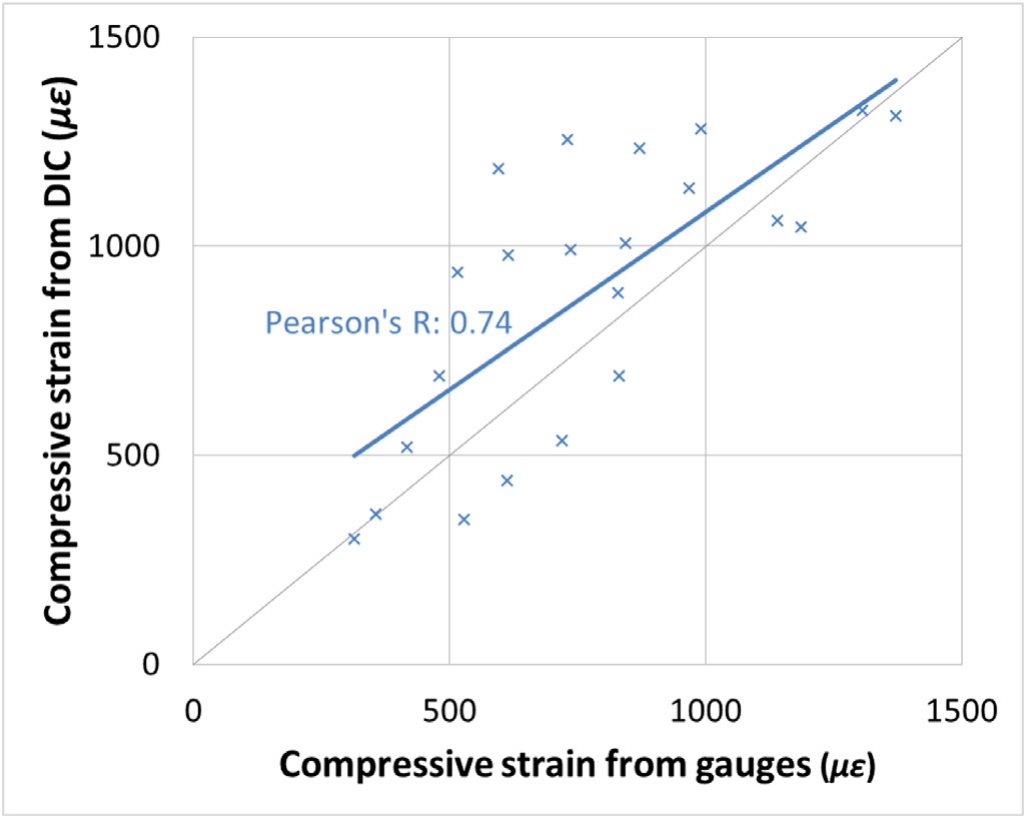
Strain–strain plot for cross-validation of DIC with strain gauges showing the correlation between the two methods.

Strain fields on each of the bone surfaces were successfully computed by the DIC algorithm and the DIC protocol produced strains that correlated (*R* = 0.74) with those measured by strain gauges in immediately adjacent bone (Fig. 3). The root mean squared difference between adjacent measurements was 260 µε.

**Fig. 4:**
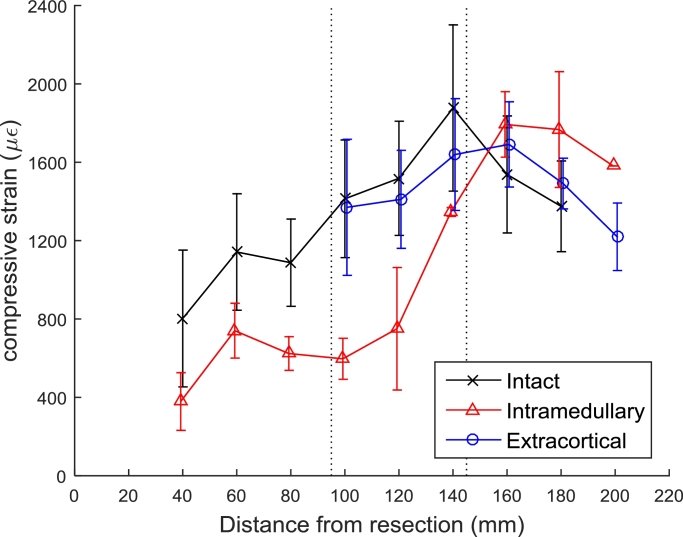
Longitudinal compressive strain on the medial surface (mean + /− SD, *n* = 4 per group).

For medial surface strains, the effect of different implant states on the measured surface strain was dependent on the distance from the tibial plateau resection (*p < 0.05*). For the cemented intramedullary implant compared to an intact bone, there was less compressive strain measured in the proximal and middle regions (*p < 0.05*, Table 2); i.e., the cemented intramedullary implant strain-shielded the cortical bone (Fig. 4 and Table 3). There was also a decrease in compressive strain in the middle region for the cemented intramedullary implant compared to the specimens which received an extracortical implant (*p < 0.05*, Table 2 and Fig. 4).

**Table 2:** Mean differences in medial cortical strains (with 95% confidence intervals and *p*-values) measured at varying distances from the tibial plateau resection with a stair climbing load distributed 70% medially.

Bone region	Distance from tibial resection (mm)	Mean difference in compressive strain (µε)		
		Intact –	Intact –	Extracortical –
		Intramedullary	Extracortical	Intramedullary
**Proximal**	20	**–**	**–**	**–**
	40	424 (CI: 62–785, *p* = 0.022)	–	–
	60	402 (CI: 40–763, *p* = 0.03)	–	–
	80	464 (CI: 102–825, *p* = 0.013)	–	–
**Middle**	100	817 (CI: 372–1260, *p* < 0.001)	43 (CI: –401–488, *p* = 1)	773 (CI: 328–1220, *p* < 0.001)
	120	768 (CI: 323–1210, *p* < 0.001)	107 (CI: –337–552, *p* = 1)	661 (CI: 216–1110, *p* = 0.002)
	140	531 (CI: 50–1010, *p* = 0.026)	237 (CI: –207–682, *p* = 0.582)	293 (CI: –187–774, *p* = 0.415)
**Distal**	160	−255 (CI: –700–189, *p* = 0.489)	−153 (CI: –598–291, *p* = 1)	−101 (CI: –547–343, *p* = 1)
	180	−391 (CI: –837–53, *p* = 0.102)	−116 (CI: –597–364, *p* = 1)	−275 (CI: –756–205, *p* = 0.49)
	200	–	–	−361 (CI: –952–228, *p* = 0.225)

**Table 3:** Percentage values of strain-shielding caused by the presence of either a cemented intramedullary implant or an extracortical implant (relative to an intact state), displayed under a variety of loading conditions.

Surface	Load case	Medial load weighting (%)	Mean ± SD strain-shielding (%)			
			Intramedullary component		Extracortical component	
			Proximal region	Middle region	Proximal region	Middle region
**Medial**	Gait (3.0BW = 2030 N)	70	45 ± 37	47 ± 29	N/a	8 ± 27
		50	38 ± 31	37 ± 25	N/a	−8 ± 48
	Stair ascent (3.6 BW = 2470 N)	70	44 ± 35	46 ± 27	N/a	8 ± 26
		50	49 ± 32	40 ± 26	N/a	−7 ± 42
**Posterior**	Gait (3.0 BW = 2030 N)	70	47 ± 13	56 ± 14	23 ± 29	19 ± 29
		50	41 ± 20	47 ± 17	22 ± 22	17 ± 17
	Stair ascent (3.6 BW = 2470 N)	70	44 ± 12	53 ± 13	27 ± 30	17 ± 27
		50	39 ± 18	46 ± 15	19 ± 20	15 ± 16

**Fig. 5:**
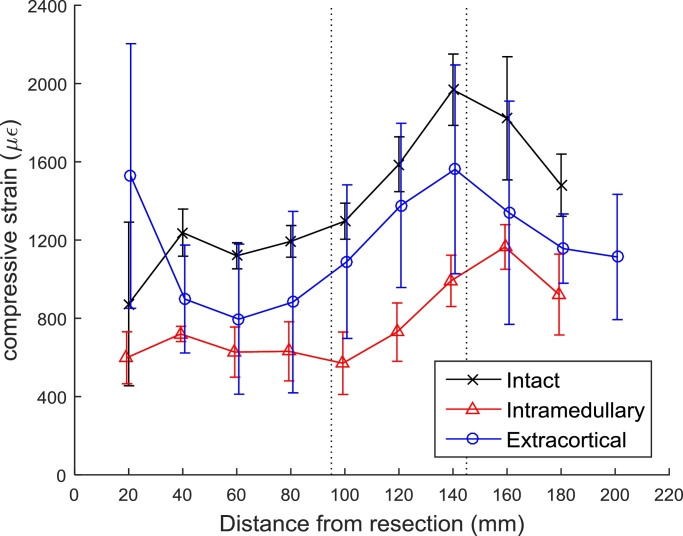
Longitudinal compressive strain on the posterior surface (mean + /− SD, *n* = 4 per group).

For posterior surface strains, the effect of different implant states on the measured surface strain was not found to be dependent on the distance from the tibial plateau resection, however there was an effect of implant state on surface strains (*p < 0.05*). Both the cemented intramedullary and extracortical implants reduced the compressive strain measured on the posterior surfaces when compared to the intact tibiae (*p < 0.05*, Table 4 and Fig. 5). However, use of a cemented intramedullary implant also resulted in reduced posterior surface strains when compared to tibiae implanted with extracortical implants (*p < 0.05*, Table 4 and Fig. 5).

**Table 4:** Mean differences in posterior cortical strains (with 95% confidence intervals and *p*-values) measured at varying distances from the tibial plateau resection with a stair climbing load distributed 70% medially.

Bone region	Distance from tibial resection (mm)	Mean difference in compressive strain (µε)		
		Intact –	Intact –	Extracortical –
		Intramedullary	Extracortical	Intramedullary
**All**	20–200	625 (CI: 449–801, *p* < 0.001)	223 (CI: 48–399, *p* = 0.008)	402 (CI: 225–578, *p* < 0.001)

For an average specimen when stair climbing with a 70% medially weighted load, the cemented intramedullary component strain-shielded the bone more than the extracortical component (for the medial surface 46% versus 8% in the middle region; for the posterior surface 44% versus 27% in the proximal region, and 53% versus 17% in the middle region, *p < 0.05*). For strain-shielding results for all load cases (Table 3), the cemented intramedullary implant strain-shielded the cortical bone more than the extracortical implant.

## 4 Discussion

The most important finding of this study was – as was hypothesised in the introduction – that an extracortical-fixation implant design reduced the amount of strain-shielding compared to conventional RTKA tibial trays with cemented intramedullary fixation stems. This result is unsurprising for the region of bone distal to the fixation plates where the cemented intramedullary implant continued to affect the bone surface strains due to its greater length. However, more relevant to the study aims, the extracortical fixation also reduced the strain-shielding in the bone adjacent to the tibial tray (the region in which associated bone resorption often leads to component loosening): the cemented intramedullary stem shielded the bone from approximately 40% of longitudinal strain, while the component with extracortical fixation shielded only 20 % (in the proximal and middle regions intact strains ranged from 800 to 2000* *µε, extracortical fixation reduced this strain by 200–400* *µε, and cemented intramedullary fixation by 400–800* *µε, Figs. 4 and 5, and Table 3). These results indicate that the primary advantage conceived for the use of extracortical plate fixation rather than cemented intramedullary stems – that of reduced strain-shielding – is valid, providing that internal cancellous bone strains do indeed correlate with bone surface strains. Given that the axial compressive bone surface strains were reduced significantly around the intramedullary stem fixation, it implies that the load was carried partly by the implant fixation stem, so that the cancellous bone in the epiphyseal zone would be subjected to less than normal loading/strain. That could be expected to lead to bone loss near the joint line. Therefore, a surgical technique utilising extracortical fixation could have an impact on outcomes of revision knee arthroplasty by reducing long-term bone loss.

To assess the implications of these findings, the relationship between strain-shielding and measured physical effects must be considered: shielding of 30–50% of the strain in cancellous bone has been shown to lead to bone resorption [35], [36], [37]. In the present study, the magnitude of strain-shielding measured in the bone surface for the implant with extracortical plates was less than this critical range (ranging −8–27%, Table 3). Assuming that strain-shielding in cancellous bone has a strong association with strain-shielding in adjacent regions of cortical bone, this study implies that use of extracortical fixation is unlikely to lead to bone resorption, which may lead to improved bone preservation when compared with the conventional design. However, the considerable differences in strain-shielding patterns between cemented intramedullary and extracortical fixation methods observed must be corroborated by additional in-vivo evidence.

Several authors have reported strain-shielding in tibiae with similar cemented stems to those in the present study, of which two provide appropriate data for comparison [34], [38]. Completo et al. [34] assessed strain-shielding with synthetic bone models similar to those used in the present study, strain gauges mounted on all surfaces, and medially weighted loading corresponding to the peak load during gait. They found strain-shielding of their cemented stem (90 mm in length) to be 45% and 47% on the posterior and medial surfaces, respectively, for gauges 53 mm from the resection plane. These values match well with the present results at a distance of 60 mm (41% and 41%, respectively). At 133 mm, their strain gauges recorded strain-shielding of 25% and 29% on the posterior and medial surfaces, respectively, which also match well with the present results at a distance of 140 mm (40% and 28%, respectively). Bourne and Finlay [38] performed a similar study with six cadaveric tibiae, applying a load of 1.47 kN, comparing a 150 mm stem with an intact case. Their strain gauges recorded much greater strain on the lateral surface when compared with the medial surface, and so it is presumed that their compressive loading was applied at a more lateral position compared with that in the present study, so it is difficult to compare strain-shielding from Bourne and Finlay's work with the present study. However, the strain-shielding was very similar. At Bourne and Finlay's third strain gauge (which may correspond to 80 mm distal to the resection plane), strain-shielding on the lateral surface was approximately 40%. At their fourth strain gauge (which may correspond to ∼140 mm distal to the resection plane), the strain-shielding was approximately 35%. Both of these results, as well as the overall shapes of both the cemented intramedullary-stemmed and intact compressive strain patterns, show excellent agreement with the results of the present study.

Both Completo et al. and Bourne and Finlay's studies [34], [38] used strain gauges whilst the present study used DIC. Strain gauges only provide single point readings and thus can be misleading in areas of high strain gradients. They also require careful surface preparation to guarantee a strong bond between the gauge and the surface. DIC measurements provide full-field strain measurements and are therefore more applicable in regions of high strain gradient but can be sensitive to speckle pattern design and the system setup. In the present study, strain gauge measurements were compared to adjacent DIC measurements to test correlation between the two methods when measuring tibial strains. An exact match was not expected because readings were taken in adjacent regions (simultaneous measurement in the same location was not possible because the gauge obscured sight of the bone), however correlation (*R* = 0.74, Fig. 3) between the two methods demonstrate they both can provide strain-shielding information, with DIC having the inherent advantage of providing readings over the entire cortical bone surface.

Several limitations of this study must be acknowledged. First, synthetic composite bones have mechanical properties similar to normal healthy bone [39], rather than the bone of elderly patients who require RTKA. In patients with osteoporotic bone, the unicortical fixation used for the locking plates may not be appropriate due to the thin cortical wall of the metaphysis. Also, the absolute values of strain measured in this experiment are likely to have been lower than those experienced in osteoporotic/osteopenic elderly bone. However, the difference in mechanical properties between the implant and bone (which causes the strain-shielding) is far greater than the difference in mechanical properties between young and elderly bone, and hence, these synthetic bones provide a suitable medium for a comparative study of implant strain-shielding. Moreover, synthetic bones have low geometric and mechanical property inter-specimen variability [39]; this is advantageous for studies where a repeated-measures design is not possible (such as the present study where the implants necessitate different bone preparation). Other authors have previously used synthetic bones to investigate bone strains following TKA/THA for these reasons [27], [40]. Second, there were small deviations in the position and orientation of surgical cuts, thus the alignments of the bones were not identical. However, this is representative of typical surgical variability and was accounted-for by testing four specimens for each implant condition. Third, with only four samples per group, differences could be subject to a type II error. However, within the proximal and middle regions, where bone loss attributed to strain-shielding is most commonly seen clinically, four samples were sufficient to reject the null hypothesis for nearly all comparisons leaving little scope for type II errors. Fourth, 6 mm machine screws were used for prototyping convenience for this proof of concept study; future work will need to convert these to conventional orthopaedic locking screws. Fifth, a moderate bone loss was simulated. The performance of the extracortical plated design should be also being investigated for cases with more severe bone loss (AORI type III). Sixth, one might expect there to be no differences between the implanted bones and an intact bone at distances greater than 150 mm (i.e., distal to both the intramedullary and extracortical implants); however, the values did not exactly converge. This is likely because the presence of an implant led to different proximal deformation of the bones, resulting in small variations of distal strains. Finally, the data relate only the surface strain fields, so other methods such as validated computer modelling will be appropriate in order to examine the effects in the cancellous bone within the tibia. The tests in this study were quasi-static, so it will be appropriate that tests should also be done under fatigue loading to ensure the long-term integrity of the construct when mounted on cadaveric bones. Critical examination of the limitations described above suggests that, because the bone models would have been stiffer than osteoporotic tibiae at RTKA, coupled with clinical cases sometimes having larger bone defects, the differences in bone surface strains reported in this paper may have under-estimated the changes in clinical reality.

The clinical benefits and risks associated with the extracortical fixation method must also be identified and understood. The clinical concerns are the requirement for additional intra-operative damage to the superficial soft tissues, which may have been compromised by previous surgery, and the possibility of longer operative time than with cemented intramedullary fixation, which may increase the likelihood of infection. However, many RTKA patients will have good soft tissue coverage, the use of extracortical plate and screw fixation has been proven [17], [18], [19], and the extraosseous design will enhance bone preservation and grafting of cavities. Further development could optimise plate positioning for soft tissue coverage and develop a minimally-invasive insertion method. This design concept may also be applied to the distal femur, with a platform supporting a conventional femoral TKA component, and soft tissue coverage would be less concerning there. Future work will also need to investigate the scope for using extracortical fixation in osteoporotic patients, when the central defect may be bone grafted [41]. Ultimately, this novel design and associated surgical technique should be deemed suitable for specific patient or condition characteristics, and may lead to enhanced bone preservation.

## 5 Conclusions

A proximally fixating extracortical plate system reduced tibial strain-shielding compared to the current intramedullary stemmed standard treatment option for RTKA. The potential for enhanced bone preservation warrants further development given the escalating burden of RTKA.
